# Novel diagnostic and prognostic classifiers for prostate cancer identified by genome-wide microRNA profiling

**DOI:** 10.18632/oncotarget.8953

**Published:** 2016-04-23

**Authors:** Helle Kristensen, Anni R. Thomsen, Christa Haldrup, Lars Dyrskjøt, Søren Høyer, Michael Borre, Peter Mouritzen, Torben F. Ørntoft, Karina Dalsgaard Sørensen

**Affiliations:** ^1^ Department of Molecular Medicine, Aarhus University Hospital, Aarhus, Denmark; ^2^ Exiqon A/S, Skelstedet, Vedbaek, Denmark; ^3^ Institute of Pathology, Aarhus University Hospital, Aarhus, Denmark; ^4^ Department of Urology, Aarhus University Hospital, Aarhus, Denmark

**Keywords:** prostate cancer, biomarker, diagnosis, prognosis, microRNA

## Abstract

**Purpose:**

This study investigates the diagnostic and prognostic biomarker potential of miRNAs in prostate cancer (PC).

**Results:**

We identified several new deregulated miRNAs between non-malignant (NM) and PC tissue samples and between more/less aggressive PC subgroups. We also developed and validated a novel 13-miRNA diagnostic classifier with high sensitivity and specificity for PC. Finally, we trained a new 3-miRNA prognostic classifier (miR-185-5p+miR-221-3p+miR-326) that predicted time to biochemical recurrence (BCR) independently of routine clinicopathological variables in a training radical prostatectomy (RP) cohort (*n* = 126) as well as in two independent validation cohorts (*n* = 110 and *n* = 99).

**Experimental Design:**

After RT-qPCR-based profiling of 752 miRNAs in 13 NM and 134 PC tissue samples (cohort 1), we selected 93 top candidate diagnostic/prognostic miRNAs for validation in two independent patient sets (cohort 2: 19 NM and 138 PC; cohort 3: 28 NM and 113 PC samples). Diagnostic potential was assessed by ROC curve analysis and prognostic potential by Kaplan-Meier, uni- and multivariate Cox regression analyses. BCR after RP was used as endpoint.

**Conclusions:**

This is the first report of a miRNA signature with significant independent prognostic value demonstrated in three PC patient cohorts.

## INTRODUCTION

Prostate cancer (PC) is the most commonly diagnosed malignancy and the fifth leading cause of cancer-related death among males in western countries [1]. PC is diagnosed by histological inspection of prostate needle biopsies, generally indicated by an elevated serum prostate specific antigen (PSA) test and/or a suspect digital rectal examination (DRE). However, PSA has low specificity for PC while biopsies have high false negative rates and often must be repeated [2]. Moreover, currently available prognostic indicators (mainly PSA, Gleason score and tumor stage) cannot accurately predict PC aggressiveness at the time of diagnosis, which has led to overtreatment of many indolent PCs partly as a result of increased PSA testing [3, 4]. There is an urgent need for new molecular biomarkers that can improve the accuracy of PC detection and better distinguish aggressive PCs that need immediate treatment, e.g. by radical prostatectomy (RP), from non-aggressive PCs that can be safely managed by active surveillance.

In this study, we investigate the diagnostic and prognostic biomarker potential of microRNAs (miRNAs) for PC. MicroRNAs comprise an abundant class of endogenous small non-coding RNAs (~22-nt) that control gene expression at the posttranscriptional level [5]. MicroRNAs are transcribed by RNA polymerase II into long, imperfectly paired stem-loop primary miRNAs, which are further processed into hairpin-containing precursor miRNAs, exported to the cytoplasm, and cleaved into a mature ~22-nt miRNA duplex [5]. Mature miRNAs guide the miRNA-induced silencing complex (miRISC) to perfect or near-perfect complementary target mRNAs, resulting in translational inhibition or mRNA degradation [5]. It has been estimated that up to 60% of human mRNAs are regulated by miRNAs, often in a highly cell type-specific manner, and miRNAs are known to influence key cellular processes, e.g. differentiation, cell cycle progression, and apoptosis [6].

Deregulated miRNA expression has been associated with malignant transformation and tumor progression in several cancer types [6]. Thus, previous profiling studies have identified deregulated miRNAs associated with PC development and/or progression [7–11], but with little overlap in identified miRNAs [12]. Still, downregulation in PC tissue of miR-125b, miR-205, and the miR-221/222 cluster as well as upregulation of miR-21, miR-375, and the miR-200 family have been consistently reported [7–9, 11–13]. However, most earlier studies, and especially those focusing on prognostic markers, have generally used relatively small patient sample sets and/or lack sufficient independent clinical validation [14, 15].

In the present study, we used three independent PC patient cohorts to identify and validate novel diagnostic and prognostic miRNA candidate markers for PC. Initially, using a training cohort, we employed global miRNA analysis to identify differentially expressed miRNAs between non-malignant and PC tissue samples and between clinically relevant subgroups of PC. Moreover, we trained and validated two new miRNA signatures with improved diagnostic and prognostic performance as compared to single miRNAs. Thus, a novel 13-miRNA diagnostic classifier distinguished non-malignant and PC samples with very high accuracy in a training set (*n* = 147) and was successfully validated in a large independent patient sample set (*n* = 157). Furthermore, a novel 3-miRNA prognostic classifier predicted biochemical recurrence (BCR) risk after RP independently of routine clinicopathological parameters in a training cohort (*n* = 126) as well as in two independent validation cohorts (*n* = 110/*n* = 99).

## RESULTS

### Deregulated miRNA expression associated with PC development and progression

To screen for novel biomarker candidates for PC, we analyzed the expression of 752 unique mature miRNAs in a training set (cohort 1) of 13 NM, 127 clinically localized PC (RP specimens), and 7 metastatic PC tissue samples (Table 1). Several significantly deregulated miRNAs were identified between NM and PC tissue samples and between clinically relevant subgroups of PC (non-metastatic/metastatic, pT2/pT3-4, high/low Gleason score, and +/− BCR after RP; Supplementary Tables S1–S6 and text below). The 93 most promising candidate miRNAs from these comparisons (Supplementary Tables S7–S11) were selected for validation in an independent patient set (cohort 2) comprising 19 NM, 113 RP, and 26 MPC tissue samples (Table 1). For further validation, we used a publicly available miRNA expression dataset (cohort 3) encompassing 28 NM, 99 RP, and 14 MPC samples [16, 17] (Table 1). Thus, in addition to the specific results described below, our study provides a compendium of deregulated miRNAs in PC (Supplementary Tables S1–S11).

**Table 1:** Clinicopathological characteristics for patient cohorts

Samples	Cohort 1 (Training)			Cohort 2 (Validation)			Cohort 3 (External validation)		
	RP (*n* = 127^#^)	MPC (*n* = 7)	NM (*n* = 13)	RP (*n* = 112^#^)	MPC (*n* = 26)	NM (*n* = 19)	RP (*n* = 99)	MPC (*n* = 14)	NM (*n* = 28)
**Median Age (range)**	63 (48–72)	63 (63–77)	70 (56–83)	62 (46–72)	73 (49–91)	62 (56–80)	58 (37–83)	59 (53–79)	56 (46–67)
**Preoperative PSA, *n* (%)**									
≤ 10 ng/mL	35 (27.6)	0 (0.0)	NA	43 (38.4)	2 (7.7)	NA	76 (76.8)	6 (42.9)	NA
> 10 ng/mL	92 (72.4)	7 (100)	NA	69 (61.6)	24 (92.3)	NA	22 (22.2)	8 (57.1)	NA
Unknown	0 (0.0)	0 (0.0)		0 (0.0)	0 (0.0)	NA	1 (1.0)	0 (0.0)	NA
Mean PSA, ng/mL (range)	16 (2–49)	74 (16–100)	NA	16 (2–65)	157 (1–500)	NA	9 (1–46)	61 (0–506)	NA
**Pathological *T*-stage, *n* (%)**									
pT2a-c	78 (61.4)	0 (0.0)	NA	66 (58.9)	1 (3.8)	NA	69 (69.7)	2 (14.3)	NA
pT3a-b	49 (38.6)	5 (71.4)	NA	46 (41.1)	5 (19.2)	NA	25 (25.2)	5 (35.7)	NA
pT4	0 (0.0)	1 (14.3)	NA	0 (0.0)	20 (76.9)	NA	5 (5.1)	2 (14.3)	NA
Unknown	0 (0.0)	1 (14.3)	NA	0 (0.0)	0 (0.0)	NA	0 (0.0)	5 (35.7)	NA
**Gleason score, *n* (%)**									
< 7	60 (47.2)	0 (0.0)	NA	43 (38.4)	1 (3.8)	NA	32 (32.3)	0 (0.0)	NA
7	53 (41.7)	2 (28.6)	NA	57 (50.9)	5 (19.2)	NA	55 (55.6)	1 (7.1)	NA
8–10	14 (11.0)	5 (71.4)	NA	12 (10.7)	20 (76.9)	NA	12 (12.1)	7 (50.0)	NA
Unknown	0 (0.0)	0 (0.0)	NA	0 (0.0)	0 (0.0)	NA	(0.0)	6 (42.9)	NA
**Nodal status, *n* (%)**									
pN0	127 (100)	0 (0.0)	NA	102 (91.1)	0 (0.0)	NA	76 (76.8)	3 (21.4)	NA
pN1	0 (0.0)	2 (28.6)	NA	3 (2.6)	2 (7.7)	NA	5 (5.1)	8 (57.1)	NA
Unknown	0 (0.0)	5 (71.4)	NA	7 (6.3)	24 (92.3)	NA	18 (18.2)	3 (21.4)	NA
**Distant Metastasis, *n* (%)**									
M0	127 (100)	0 (0.0)	NA	112 (100)	0 (0.0)	NA	99 (100)	0 (0.0)	NA
M1	0 (0.0)	7 (100)	NA	0 (0.0)	26 (100)	NA	0 (0.0)	14 (100)	NA
Unknown	0 (0.0)	0 (0.0)	NA	0 (0.0)	0 (0.0)	NA	0 (0.0)	0 (0.0)	NA
**Surgical margin status, *n* (%)**									
Negative	88 (69.3)	NA	NA	80 (71.4)	NA	NA	78 (78.8)	NA	NA
Positive	38 (29.9)	NA	NA	31 (27.7)	NA	NA	21 (21.2)	NA	NA
Unknown	1 (0.8)	NA	NA	1 (0.9)	NA	NA	0 (0.0)	NA	NA
**Recurrence status, *n* (%)**									
No biochemical recurrence	70 (55.1)	NA	NA	62 (55.4)	NA	NA	74 (74.8)	NA	NA
Biochemical recurrence	57 (44.9)	NA	NA	50 (44.6)	NA	NA	25 (25.3)	NA	NA
**Mean follow-up time, months (range)**	36 (2–90)	NA	NA	40 (3–123)	NA	NA	72 (1–179)	NA	NA

Abbreviations: MPC, metastatic prostate cancer (primary tumor tissue from patient with metastatic PC); NA, not applicable/available; NM, non-malignant prostate; PSA, prostate specific antigen; RP, radical prostatectomy.

# For recurrence-free survival analyses, one RP patient from cohort 1 and 2 RP patients from cohort 2 were excluded due to postoperative endocrine treatment. Hence, the final RP cohort 1 and 2 included 126 and 110 RP patients, respectively.

In cohort 1 (training), we found 24 significantly upregulated and 45 significantly downregulated miRNAs in PC compared to NM prostate tissue samples after correction for multiple testing (Supplementary Table S2). Among 29 miRNAs (Supplementary Table S2) selected for further testing in cohort 2, we successfully validated 11 upregulated and 11 downregulated miRNAs (Supplementary Table S7), including 15 top candidate miRNAs that were at least 2-fold deregulated in both cohorts (Table 2). The most significantly upregulated miRNAs in PC were miR-375, miR-615-3p, and miR-425-5p, while the most significantly downregulated were miR-205-5p, miR-221-3p, miR-222-3p, and miR-455-3p (Table 2). Notably, we identified and validated several miRNAs not previously reported as deregulated in PC (upregulated: miR-425-5p, miR-615-3p; downregulated: miR-136-5p, miR-154-5p, miR-376c-3p, and miR-455-3p/5p; Table 2). Supporting the validity of our results, we also confirmed the up-regulation of miR-375 and the downregulation of miR-205-5p [8, 10, 18, 19], miR-221-3p [8, 11, 20–22], and miR-222-3p [11, 20–23] (Table 2), which are known hallmarks of PC [12, 14, 15]. Significant deregulation in PC vs. NM prostate tissue samples was confirmed for 14 out of 15 top candidate diagnostic miRNAs (all except miR-615-3p) in the external cohort 3 (Table 2).

**Table 2:** Successfully validated miRNAs from the comparison of non-malignant and prostate cancer samples

	Cohort 1 (13 NM *vs*. 134 PC)			Cohort 2 (19 NM *vs*. 138 PC)			Cohort 3 (28 NM *vs*. 113 PC)		
Upregulated in PC	FC	BH corrected *P* value	AUC (95% CI)	FC	BH corrected *P* value	AUC (95% CI)	FC	BH corrected *P* value	AUC (95% CI)
miR-375	3.31	**< 0.001**	0.96 (0.92–0.99)	2.46	**< 0.001**	0.83 (0.75–0.91)	2.01	**< 0.001**	0.83 (0.76–0.90)
miR-663b	111.98	**< 0.001**	0.94 (0.90–0.99)	4.37	**0.036**	0.66 (0.53–0.79)	2.67	**< 0.001**	0.86 (0.79–0.93)
miR-615-3p	26.52	**< 0.001**	0.90 (0.85–0.96)	15.47	**< 0.001**	0.83 (0.74–0.93)	−1.48	**0.117**	0.60 (0.48–0.71)
miR-425-5p	2.78	**< 0.001**	0.89 (0.83–0.96)	2.59	**< 0.001**	0.85 (0.75–0.95)	1.51	**< 0.001**	0.83 (0.76–0.90)
miR-663a	3.52	**0.001**	0.82 (0.70–0.95)	3.99	**0.006**	0.72 (0.59–0.84)	2.67	**< 0.001**	0.86 (0.79–0.93)
miR-182-5p	2.22	**0.023**	0.73 (0.62–0.85)	3.43	**< 0.001**	0.79 (0.70–0.89)	2.07	**< 0.001**	0.89 (0.83–0.95)
miR-183-5p	2.16	**0.038**	0.72 (0.62–0.81)	2.72	**0.011**	0.70 (0.58–0.82)	3.40	**< 0.001**	0.90 (0.84–0.97)
**Downregulated in PC**	**FC**	**BH corrected *P* value**	**AUC (95% CI)**	**FC**	**BH corrected *P* value**	**AUC (95% CI)**	**FC**	**BH corrected *P* value**	**AUC (95% CI)**
miR-205-5p	−22.39	**< 0.001**	0.97 (0.95–1.00)	−23.12	**< 0.001**	0.94 (0.87–1.00)	−7.38	**< 0.001**	0.86 (0.79–0.92)
miR-221-3p	−3.04	**< 0.001**	0.95 (0.90–1.00)	−3.26	**< 0.001**	0.88 (0.80–0.96)	−2.92	**< 0.001**	0.93 (0.89–0.97)
miR-222-3p	−2.95	**< 0.001**	0.92 (0.83–1.00)	−2.83	**< 0.001**	0.88 (0.81–0.95)	−3.25	**< 0.001**	0.92 (0.87–0.96)
miR-376c-3p	−3.90	**< 0.001**	0.92 (0.85–0.99)	−2.62	**0.002**	0.74 (0.63–0.86)	−1.47	**< 0.001**	0.74 (0.65–0.84)
miR-136-5p	−6.10	**< 0.001**	0.86 (0.76–0.97)	−2.40	**0.021**	0.68 (0.56–0.80)	−1.31	**0.004**	0.68 (0.58–0.79)
miR-455-3p	−2.95	**< 0.001**	0.86 (0.77–0.95)	−3.48	**< 0.001**	0.80 (0.71–0.90)	−1.56	**0.002**	0.70 (0.60–0.80)
miR-455-5p	−4.55	**0.002**	0.81 (0.69–0.93)	−3.33	**0.010**	0.71 (0.59–0.82)	−1.80	**< 0.001**	0.80 (0.72–0.88)
miR-154-5p	−3.28	**0.016**	0.75 (0.59–0.91)	−3.33	**0.010**	0.70 (0.60–0.80)	−1.32	**< 0.001**	0.76 (0.68–0.85)

Results for the top candidates with fold change > ± 2 in both the training cohort 1 and validation cohort 2 are shown. These 15 top candidate diagnostic miRNAs were also tested in the external validation cohort 3.

Furthermore, in cohort 1, we found several miRNAs to be significantly (uncorrected *P* < 0.05, Wilcoxon rank test) differentially expressed in primary tumor tissue samples from patients with metastatic vs. non-metastatic PC or from RP patients with pT3-4 vs. pT2, high (≥ 7) vs. low (< 7) Gleason score, or +/− BCR after surgery (Supplementary Tables S3–S6). Although none of these miRNAs remained significant in cohort 1 after correction for multiple testing using a strict 5% FDR, we selected the most promising candidates (based on uncorrected *P*-values and expression fold changes) for further testing in cohort 2 (Supplementary Tables S8–S11). In cohort 2, we confirmed the upregulation of miR-185-5p and the downregulation of miR-133a and miR-133b in metastatic compared to non-metastatic PC (Supplementary Table S8), which was successfully validated also in the external cohort 3 (Supplementary Table S12). We also found miR-133a to be significantly downregulated in advanced stage (pT3-4), high Gleason score, and recurrent PC in both the training cohort (cohort 1) and in the two validation cohorts (Supplementary Table S12). Likewise, in cohorts 1 and 2, miR-221-3p and miR-222-3p were significantly downregulated in high Gleason score and in recurrent tumors, whereas miR-1 and miR-204-5p were significantly downregulated in recurrent tumors only and miR-205-5p in high Gleason score tumors only (Supplementary Table S12). Although not statistically significant in all cases, the same trend was seen in cohort 3 for all of these miRNAs (Supplementary Table S12).

In total, we identified 8 miRNAs (miR-1, miR-133a, miR-133b, miR-185-5p, miR-204-5p, miR-205-5p, miR-221-3p, and miR-222-3p) associated with clinicopathological measures of PC aggressiveness in multiple cohorts (Supplementary Table S12). We note that the number of validated miRNA candidates between different PC subgroups, as well as their expression fold changes, were lower than between PC and NM tissue samples (Table 2). This is in agreement with results from previous miRNA profiling studies for PC [14, 15] and likely reflects molecular heterogeneity.

### Diagnostic performance of single miRNAs and development of a 13-miRNA diagnostic classifier

**Figure 1:**
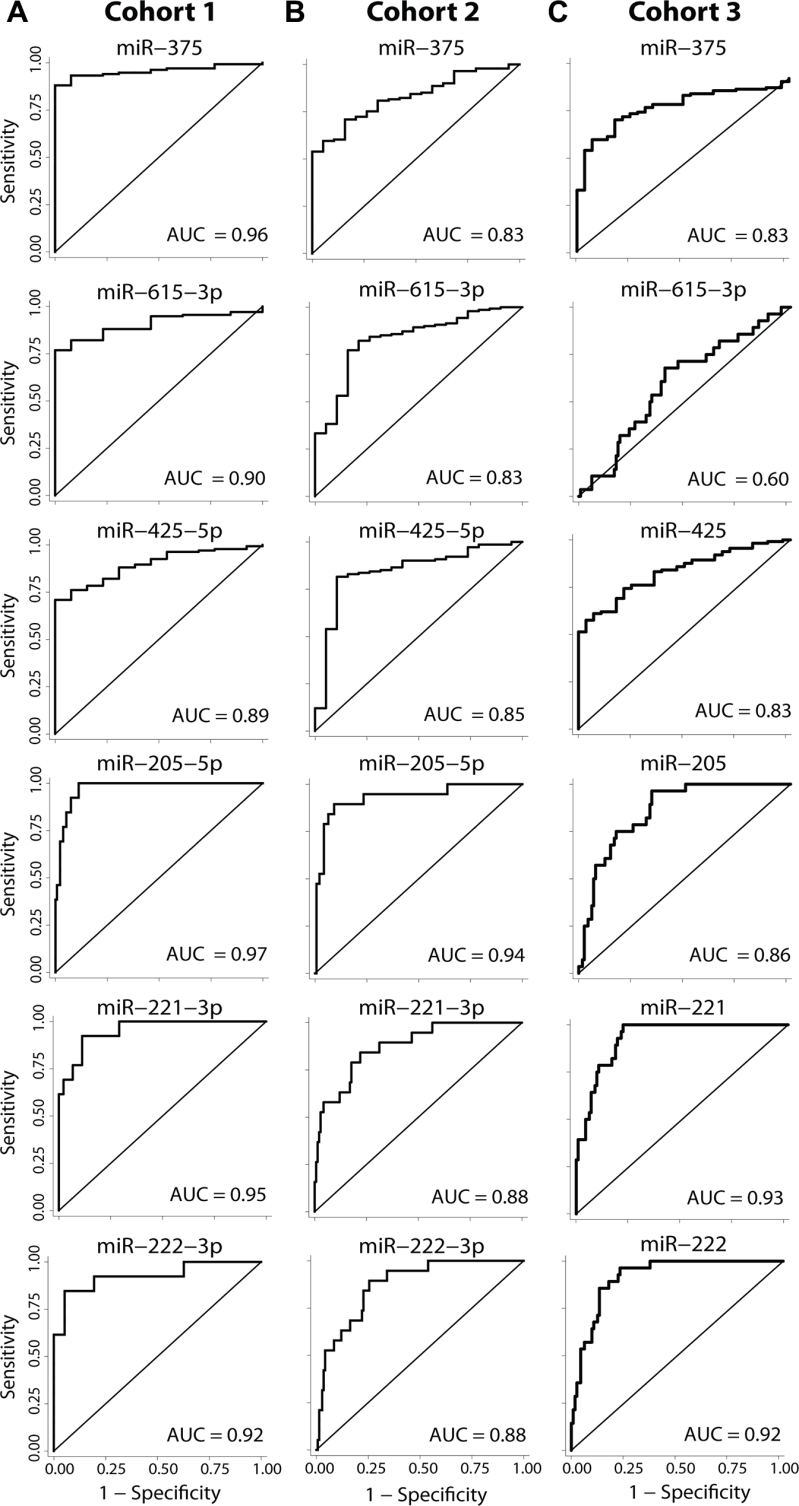
Diagnostic potential of top miRNA candidates Receiver operating characteristics (ROC) analysis of the six miRNAs with the highest diagnostic potential (AUC > 0.83) in both cohort 1 (**A**) and cohort 2 (**B**). The miRNAs are ordered as appearing in Table 2. (**C**) The diagnostic potential of the top candidates were further tested in the external data set (cohort 3). Cohort 1 consists of 13 non-malignant and 134 prostate cancer FFPE samples. Cohort 2 consists of 19 non-malignant and 141 prostate cancer FFPE samples. Cohort 3 consists of 28 non-malignant and 113 prostate cancer fresh frozen samples. AUC; area under the ROC curve.

Next, we assessed the diagnostic potential of the top 15 single miRNA candidates identified above. ROC curve analyses gave AUCs from 0.70–0.97 in cohort 1 and 0.66–0.94 in cohort 2 (Table 2). Six of these miRNAs (miR-205-5p, miR-221-3p, miR-222-3p, miR-375, miR-425-5p, and miR-615-3p) had an AUC ≥ 0.83 in both cohorts, suggesting particularly promising diagnostic potential for PC (Figure 1A–1B). Moreover, five of these miRNAs (all except miR-615-3p) also showed very good discriminative power between NM and PC samples (AUC 0.83–0.92) in the external validation cohort 3 (Table 2, Figure 1C). We note that this is the first report of a diagnostic biomarker potential for miR-425-5p in PC, as demonstrated in 3 independent cohorts (AUC:0.89/0.85/0.83; Table 2, Figure 1C).

To investigate if a miRNA signature may increase diagnostic accuracy over single miRNAs, we employed a leave-one-out cross-validation (LOOCV) maximum likelihood classifier approach [24] and trained on miRNA expression profiles from 13 NM vs. 134 PC samples in cohort 1 (Supplementary Figure S1). The final 13-miRNA diagnostic classifier (see Supplementary Table S13) included 4 of the 15 top candidate diagnostic miRNAs (miR-221-3p, miR-222-3p, miR-615-3p, and miR-663b) identified above (Table 2) and correctly classified 95.9% (*P* < 0.0001, Chi^2^ test) of NM vs. PC samples in cohort 1, corresponding to 96.3% sensitivity and 92.3% specificity (Supplementary Table S14). The 13-miRNA diagnostic classifier was independently validated in cohort 2, where it correctly classified 86.0% of 19 NM vs. 138 PC samples (*P* < 0.0001, Chi^2^ test) and showed 87.7% sensitivity and 73.7% specificity for PC (Supplementary Table S14). Notably, the discriminative power of the 13-miRNA diagnostic classifier exceeded that of each top candidate single miRNAs in cohorts 1 and 2, with the sole exception of miR-205-5p that was superior (94.3% correctly classified; sensitivity/specificity = 97.1%/73.7%) in cohort 2 but not in cohort 1 (Supplementary Table S14). The 13-miRNA diagnostic classifier could not be tested in cohort 3, as not all miRNAs were analyzed on the microarray [17]. In conclusion, we have identified a novel 13-miRNA classifier with improved diagnostic potential compared to single miRNAs.

### Prognostic performance of single miRNAs and development of a 3-miRNA prognostic classifier

To investigate the prognostic potential of miRNA expression in PC, we initially performed biochemical recurrence-free survival (RFS) analysis for 45 candidate miRNAs that were identified above as deregulated in pT2 *vs.* pT3-4, low *vs.* high Gleason score, and/or recurrent *vs.* non-recurrent tumors in cohort 1 (Supplementary Tables S4–S6). By univariate Cox regression analysis, 11 of these miRNAs were significantly associated with RFS time in RP cohort 1 (*n* = 126) (Supplementary Table S15; uncorrected *P* < 0.05, and FDR < 0.20 after correction for multiple testing). More specifically, high expression of miR-10b-5p, miR-23a-3p, miR-185-5p, miR-615-3p, and miR-625-3p and low expression of miR-30d-3p, miR-133a, miR-193a-5p, miR-221-3p, miR-326, and miR-374b-5p was associated with early BCR in this cohort. However, none of these 11 individual miRNAs remained significant in a multivariate model including routine clinicopathological factors (PSA, pT stage, Gleason score, and margin status; data not shown). Moreover, their prognostic value in univariate analysis in RP cohort 1 could generally not be confirmed in the independent RP cohort 2 (*n* = 110) or RP cohort 3 (*n* = 99) (Supplementary Table S15).

**Figure 2:**
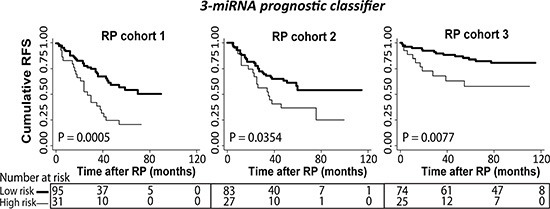
Kaplan-Meier plots with prostate-specific antigen recurrence as end point Kaplan-Meier survival analysis of recurrence free survival (RFS) based on the 3-miRNA prognostic classifier (miR-185-5p+miR-221-3p+miR-326) in prostatectomy samples from three independent RP cohorts. Patients in the training RP cohort 1 (left) were divided into low *vs.* high risk groups after ROC analysis. Patients in validation RP cohort 2 (middle) were divided into low/high risk groups according to the cut-off (fraction) defined in RP cohort 1. This was done in the same way for the external validation RP cohort 3 (right). Significant *P* values for two-sided log-rank test are given. High molecular risk status, as defined by the 3-miRNA prognostic classifier (miR-185-5p+miR-221-3p+miR-326), was significantly associated with early biochemical recurrence after RP in three independent cohorts.

Instead, we investigated if a combination of the 11 miRNAs might improve RFS time prediction over single miRNAs. Thus, using combined weighed sum models and a stepwise exclusion approach, we trained a novel 3-miRNA prognostic classifier (miR-185-5p+miR-221-3p+miR-326) that was significantly associated with RFS in univariate Cox regression analysis in RP cohort 1 (*P* < 0.001) and remained significant (*P* = 0.031) also in a multivariate model adjusted for pT stage, Gleason score, surgical margin status, and preoperative PSA (Table 3). The significant prognostic value of the 3-miRNA prognostic classifier in univariate as well as multivariate Cox regression was independently validated in both RP cohort 2 and RP cohort 3 (Table 3). Notably, addition of the 3-miRNA prognostic classifier to a multivariate model including clinicopathological factors only, increased the predictive accuracy (estimated by Harrell's C-index) from 0.72 to 0.74 in RP cohort 1, from 0.73 to 0.75 in RP cohort 2, and from 0.74 to 0.80 in RP cohort 3 (Table 3), indicating improved performance. Furthermore, Kaplan-Meier analyses showed a significant association between the 3-miRNA prognostic classifier and RFS in RP cohort 1 (*P* = 0.0005, log-rank test), which was successfully validated in RP cohort 2 (*P* = 0.0354) as well as in RP cohort 3 (*P* = 0.0077) (Figure 2). In summary, we have trained, tested, and validated a novel 3-miRNA prognostic classifier (miR-185-5p+miR-221-3p+miR-326) that predicted time to BCR after RP independently of routine clinicopathological parameters in three independent PC patient cohorts.

**Table 3:** Prognostic potential of the 3-miRNA prognostic classifier (miR-185-5p+miR-221-3p+miR-326) assessed by uni- and multivariate Cox regression analyses of biochemical recurrence-free survival time in three RP cohorts

Variable	Characteristics	Univariate			Multivariate^c^			
		HR (95% CI)	*P* value	C-index^a^	HR (95% CI)	*P* value	C-index^b^	
**RP cohort 1, *n* = 126, 56 with recurrence**								
Age at diagnosis	Continuous	1.00 (0.94–1.05)	0.858	0.53	–	–		
Tumor stage	pT2a-c vs. pT3a-c	3.12 (1.81–5.36)	**< 0.001**	0.64	–	–		
Gleason score	< 7 vs.≥ 7	2.73 (1.51–4.93)	**0.001**	0.61	2.68 (1.46–4.93)	**0.001**	0.72	0.74
Surgical margin status	Negative vs. positive	2.73 (1.59–4.70)	**< 0.001**	0.63	2.46 (1.39–4.34)	**0.002**		
Preoperative PSA	Continuous	1.05 (1.02–1.08)	**< 0.001**	0.62	1.04 (1.01–1.07)	**0.005**		
3-miRNA classifier^d^	Continuous	1.71 (1.31–2.24)	**< 0.001**	0.66	1.36 (1.03–1.79)	**0.031**		
**RP cohort 2, *n* = 110, 49 with recurrence**								
Age at diagnosis	Continuous	0.97 (0.93–1.03)	0.319	0.53	–	–		
Surgical margin status	Negative vs. positive	3.37 (1.89–6.00)	**< 0.001**	0.64	–	–		
Gleason score	< 7 vs.≥ 7	2.42 (1.23–4.73)	**0.010**	0.59	–	–		
Tumor stage	pT2a-c vs. pT3a-c	3.00 (1.69–5.30)	**< 0.001**	0.63	3.21 (1.76–5.84)	**< 0.001**	0.73	0.75
Preoperative PSA	Continuous	1.05 (1.03–1.07)	**< 0.001**	0.72	1.05 (1.02–1.07)	**< 0.001**		
3-miRNA classifier^d^	Continuous	1.44 (1.11–1.88)	**0.006**	0.58	1.28 (1.00–1.64)	**0.048**		
RP cohort 3, *n* = 99, 25 with recurrence^*^								
Age at diagnosis	Continuous	1.03 (0.98–1.09)	0.278	0.56	–	–		
Tumor stage	pT2a-c vs. pT3a-c	4.05 (1.80–9.12)	**0.001**	0.68	–	–		
Surgical margin status	Negative vs. positive	3.81 (1.70–8.54)	**0.001**	0.63	2.40 (0.94–6.12)	**0.007**	0.74	0.80
Preoperative PSA	Continuous	1.09 (1.06–1.13)	**< 0.001**	0.66	1.06 (1.02–1.11)	**0.008**		
3-miRNA classifier^d^	Continuous	2.10 (1.42–3.10)	**< 0.001**	0.70	1.91 (1.26–2.91)	**0.012**		

Abbreviations: CI, confidence Interval; HR, hazard ratio; PSA, prostate specific antigen; pT, pathological tumor stage; RP, radical prostatectomy.

a Predictive accuracy estimated by Harrell's concordance index (C-index).

b Left column, C-index based on clinicopathological variables only (i.e. excluding miRNA classifier expression); right column, C-index based on all variables included in the model.

c The 3-miRNA prognostic classifier was analyzed in multivariate analysis including tumor stage, Gleason score, surgical margin, and preoperative PSA. In the final multivariate model, variables failing the global multivariate analysis were excluded by stepwise backward selection.

d For generation of this 3-miRNA prognostic classifier, a weighted sum was calculated. The expression level of each miRNA was weighed by the estimated regression coefficients in a multivariate proportional hazards model (trained in RP cohort 1, and tested in RP cohorts 2 and 3).

* Gleason score was excluded from analysis in RP cohort 3, because the low Gleason score group (< 7) had no events. Significant *P* values (*P* < 0.05) are marked in bold.

## DISCUSSION

This work represents one of the largest and most comprehensive miRNA expression profiling studies of PC to date. We identified several miRNAs that were significantly deregulated in PC compared to NM prostate tissue samples and/or between clinically relevant subgroups of PC. In addition, we built and validated a new 13-miRNA diagnostic classifier that distinguished PC from NM prostate tissue samples with very high accuracy in two independent patient sets. We also developed a new 3-miRNA prognostic classifier (miR-185-5p+miR-221-3p+miR-326) that predicted BCR after RP in three PC patient cohorts independently of routine clinicopathological variables.

Based on the hypothesis that miRNA classifiers may improve sensitivity and specificity over single markers, we trained and validated a new 13-miRNA diagnostic classifier. The diagnostic accuracy of this classifier was superior to all single candidate miRNAs investigated, except for miR-205-5p in cohort 2, suggesting it is more robust across a range of PC cohorts. The performance of our 13-miRNA diagnostic classifier (96% and 86% correct classification in cohort 1 and 2, respectively) is comparable to that of a 54-miRNA classifier developed earlier by Martens-Uzunova *et al*. [20]. Six miRNAs (miR-21-3p, miR-27b, miR-30c, miR-93-5p, miR-221-3p, and miR-222-3p) are shared between these classifiers, further supporting the validity of our findings.

Although the relatively small number of NM as compared to PC samples is a potential limitation for the diagnostic part of our study, miRNA expression patterns were more homogeneous in the non-malignant samples (data not shown), justifying this study design. Furthermore, our results were based on analyses of RP specimens, whereas a future diagnostic test for PC should use more clinically relevant sample types, such as urine or blood. Elevated miR-615-3p [25] and reduced miR-205 levels [26] have been reported in urine samples from PC patients compared to controls, whereas increased miR-375 levels have been found in serum and plasma samples from PC patients [12, 27]. Further studies are needed to investigate whether our top candidate single miRNAs (Table 2) and/or our 13-miRNA diagnostic classifier may be transferred to a urine or blood-based test [28].

Even though we did not find any single miRNA with significant independent prognostic value in our three RP cohorts, we successfully validated a novel 3-miRNA prognostic classifier (miR-185-5p+miR-221-3p+miR-326). This suggests that multi-miRNA classifiers are superior to single miRNAs for PC prognostication. Indeed, although PC samples in cohort 3 were snap-frozen (rather than FFPE), sampled in another country, and analyzed on a distinct miRNA platform, our 3-miRNA prognostic classifier performed well also in this cohort, suggesting that it is very robust.

Of the 3 miRNAs included in our validated prognostic classifier, only miR-221 has previously been included in prognostic miRNA signatures for PC, but the earlier studies all lacked independent validation and/or multivariate analysis [20, 21, 29]. One of the studies [21] presented a model called miQ (miR-96-5p, miR-183-5p, miR-145-5p, and miR-221-5p) that distinguished aggressive from non-aggressive PC in a training set (*n* = 49) and was validated using data from Taylor *et al.* [16, 17], as also used here. This further supports a prognostic potential of miR-221 in relation to PC. Although miR-221 was not a significant independent predictor of time to BCR in the present study as a single marker, low miR-221 expression has previously been associated with BCR in multivariate analysis in one RP cohort (*n* = 118) [30] and with clinical failure in multivariate analysis in another RP cohort (*n* = 92) [22]. Moreover, overexpression of miR-221 in PC cell lines inhibits growth and invasion and stimulates apoptosis [31], indicating that miR-221 has tumor suppressor functions in PC.

There are no previous reports of a prognostic potential in PC for the remaining miRNAs in our 3-miRNA prognostic classifier. Similar to our current finding, low expression of miR-326 has been linked with poor prognosis in glioma [32], colorectal [33], and pancreatic cancer [34]. Moreover, miR-326 has been found to inhibit proliferation, migration, and invasion of colorectal cancer cell lines [33], but no PC related functions have been reported. Furthermore, whereas high miR-185-5p expression was associated with adverse prognosis in our RP cohorts, previous studies have shown that miR-185 suppresses proliferation, migration, and invasion and induces apoptosis in some PC cell lines [35–37]. This seeming inconsistency may be explained by cell type-specific differences, as has been demonstrated for several other miRNAs that function as oncogenes in some cells and as tumor suppressor in others [6]. Also, the earlier reports [35–37] did not distinguish between miR-185-5p and miR-185-3p.

We are the first to report a miRNA based prognostic biomarker signature (or single miRNA) with significant independent prognostic value in three PC patient cohorts. A possible limitation of our study is the use of BCR as clinical endpoint. Furthermore, the BCR rate in RP cohorts 1 and 2 was relatively high (approx. 45%; Table 1), hence a possible selection bias for larger tumors cannot be ruled out. However, our prognostic signature was validated also in cohort 3 with a lower (25%; Table 1) BCR rate, similar to that of contemporary RP cohorts. Future studies should investigate other and more clinically relevant endpoints, such as metastatic progression, cancer-specific and overall mortality. Due to the slow disease course of PC this would require large cohorts with > 15 years of follow-up [38].

Another potential limitation of our study is the use of post-operative tissue specimens, as there are currently no established adjuvant therapies for patients recurring after RP. Still, given the current evidence, patients who are scored as having high risk of BCR based on our 3-miRNA prognostic classifier, could be candidates for e.g. adjuvant radiation therapy [39]. An important future task will be to investigate if our 3-miRNA prognostic classifier can predict PC aggressiveness at the time of diagnosis based on analysis of prostate biopsies (or even a urine or blood sample) and hence be used to guide treatment decisions, e.g. active surveillance vs. surgery.

In conclusion, we have shown that the combination of multiple miRNAs into molecular classifiers may improve the diagnostic and prognostic biomarker potential of miRNAs for PC. In order to translate our new diagnostic and prognostic miRNA classifiers into potential future clinical use, they must be further validated in multi-center studies using large, well-characterized patient cohorts and clearly defined clinical endpoints. Future studies should also assess their potential for non- or minimally invasive testing in urine and blood samples.

## MATERIALS AND METHODS

### Clinical samples and RNA extraction

For miRNA profiling by RT-qPCR, we used formalin-fixed paraffin-embedded (FFPE) prostate tissue samples from two distinct patient sample sets (Table 1; cohorts 1 and 2). Cohort 1 (training) consisted of 13 non-malignant (NM) prostate tissue samples from benign prostatic hyperplasia (BPH) patients, 127 clinically localized PC tissue samples from curatively intended RPs, and 7 primary tumor samples from metastatic PC (MPC) patients. Cohort 2 (validation) consisted of 19 adjacent NM prostate tissue samples, 112 clinically localized PC tissue samples from curatively intended RPs, and 26 primary tumor samples from MPC patients. All samples were collected (1997–2005) at Department of Urology and obtained from Institute of Pathology, Aarhus University Hospital, Denmark. The study was approved by the local scientific ethical committee and by the Danish Data Protection Agency. Written informed consent was obtained from all patients.

Prior to RNA extraction, all tissue specimens were evaluated by one highly experienced histopathologist (chief pathologist, Dr. Søren Høyer). Gleason scoring was performed according to ISUP 2014 criteria [40], representative areas with > 90% tumor were marked on hematoxylin and eosin (H&E) stained sections, and 1.5 mm punch biopsies were taken from the corresponding FFPE blocks, as described previously [41]. Total RNA was isolated from punch biopsies using the miRNeasy FFPE Kit (Qiagen). RNA samples with 260/280 nm absorbance ratio < 1.75 were excluded from further analysis. Adjacent NM samples from RP specimens and BPH and MPC samples from transurethral resections of the prostate were processed in the same way.

A flow chart of inclusion/exclusion criteria according to REMARK guidelines is shown in Supplementary Figure S2. For biochemical recurrence-free survival (RFS) analyses, RP samples from cohorts 1 and 2 were used. These sample sets have previously been used for tissue microarray (TMA) construction [41, 42]. For RP cohort 1, we could retrieve FFPE tissue blocks and extract RNA of sufficient quality for 177 out of 196 patients included on the TMA (Supplementary Figure S2A). Only patients with urine, plasma, and serum samples available (separate study) were further selected for RP cohort 1 (*n* = 136). Another 9 patients were excluded due to either pre-operative endocrine treatment (*n* = 2), lack of follow-up (*n* = 3), or failed miRNA analysis (*n* = 4). Of the remaining 127 RP samples, 126 were used for RFS analysis (one patient excluded due to post-operative endocrine treatment; Table 1). For RP cohort 2 (validation), inclusion/exclusion criteria were employed as for RP cohort 1, except for the matching biofluid requirement (Supplementary Figure S2A). Of the remaining 112 RP samples, 110 were used for RFS analysis (two patients excluded due to post-operative endocrine treatment; Table 1).

For external validation (cohort 3, Table 1), we used publicly available Agilent Human miRNA Microarray 2.0 expression data (GSE21036) for 368 miRNAs in 28 adjacent NM, 99 RP, and 14 MPC snap-frozen prostate tissue samples. Based on histological assessment, RNA from the PC samples in cohort 3 was extracted using areas with > 70% tumor cells. The exact version of the Gleason grading system used was not specified [16, 17].

### MicroRNA profiling

MicroRNA expression profiling (all reagents from Exiqon) was performed at Exiqon A/S, Vedbaek, Denmark, using the miRCURY LNA^™^ Universal RT microRNA PCR platform. For cohort 1 (training), relative expression levels of 752 miRNAs were analyzed using microRNA Ready-to-Use PCR, Human panel I + II, V3.R, in 384-well PCR plates. For cohort 2 (validation), 94 selected miRNAs (including normalization gene miR-151a-5p) were analyzed using a miRCURY LNA™ Universal RT Pick-&-Mix microRNA PCR panel (4 × 96 in 384-well, Ready-to-Use). For further information, see Supplementary Methods.

### Statistical analyses

Unless stated otherwise, statistical analyses were conducted in STATA version 11 (StataCorp, Texas, USA). *P* values < 0.05 were considered significant. The Wilcoxon signed-rank test was used for pairwise comparisons of miRNA expression levels between sample subgroups. Only miRNAs expressed in more than 70% of the samples in each subgroup were included in the analyses. *P* values were corrected for multiple testing using the Benjamini-Hochberg method [43]. The diagnostic potential of miRNA expression was evaluated by receiver operator characteristics (ROC) curve analysis. Furthermore, normalized miRNA expression values from 13 NM and 134 PC samples (cohort 1, training) were used to construct a diagnostic miRNA classifier. Only miRNAs expressed in at least 70% of the samples were used (*n* = 235 miRNAs). Maximum likelihood classification procedures were trained and tested as described previously [24].

For RFS analyses, biochemical recurrence (BCR; PSA cut-off ≥ 0.2 ng/ml) was used as endpoint. Patients not having experienced BCR were censored at their last normal PSA test. The prognostic value of miRNA expression was evaluated by Kaplan-Meier analysis, two-sided log-rank tests, and by uni- and multivariate Cox regression analyses.

For training of the prognostic miRNA classifier, we used the 11 miRNAs that were significant in univariate Cox regression analysis in the training cohort. Each miRNA included in the classifier was weighed by the estimated regression coefficients in the multivariate Cox proportional hazards model, and a combined weighted sum for the miRNA classifier was calculated. For analysis of miRNA/classifier expression as a dichotomous variable, patients in RP cohort 1 (training) were divided into high and low expression groups using a cut-off value determined by ROC analysis of BCR status. For independent validation, patients in RP cohorts 2 and 3 were dichotomized into low/high expression groups using the cut-off (fraction) defined in RP cohort 1. All clinicopathological parameters significant in univariate analysis were included in multivariate analyses. Variables failing multivariate analysis were excluded from the final multivariate model through stepwise backward selection. The proportional hazards assumption was verified by the log-negative-log survival distribution function for all variables. Prognostic accuracy was estimated using Harrell's Concordance Index (C-index).

## SUPPLEMENTARY MATERIALS TABLES AND FIGURES


