# Supplementary material for: Novel diagnostic and prognostic classifiers for prostate cancer identified by genome-wide microRNA profiling
Source: Oncotarget. 2016 Apr 23;7(21):30760–71. doi: 10.18632/oncotarget.8953 (PMC5058715; doi:10.18632/oncotarget.8953)
Supplement: Supplementary file 1 [file oncotarget-07-30760-s001.pdf]

Novel diagnostic and prognostic classifiers for prostate cancer identified by genome-wide microRNA profiling

Supplementary Materials

Supplementary Table S1: Number of samples included in pairwise comparisons for each of the three cohorts

Pairwise comparison		Cohort 1		Cohort 2		Cohort 3	
		Group 1	Group 2	Group 1	Group 2	Group 1	Group 2
Diagnostic	NM *vs.* PC	13	134	19	138	28	113
Disseminated	Non-metastatic *vs.* metastatic PC	127	7	112	26	99	14
Tumor stage	pT2 *vs.* pT3-4	78	49	66	46	69	30
Gleason score	< 7 *vs.*≥ 7	60	67	43	69	32	67
Prognostic	No BCR *vs.* BCR	70	57	62	50	74	25

Abbreviations: NM, non-malignant; PC; prostate cancer; BCR, biochemical recurrence after radical prostatectomy.

Supplementary Table S2: List of significantly deregulated miRNAs in NM (*n* = 13) *vs.* PC samples (*n* = 134) in cohort 1

Upregulated in PC	FC	*P* value	BH corrected *P* value	Downregulated in PC	FC	*P* value	BH corrected *P* value
miR-375[§]	3.31	**< 0.001**	**< 0.001**	miR-205-5p[§]	−22.39	**< 0.001**	**< 0.001**
miR-200c-3p[§]	2.74	**< 0.001**	**< 0.001**	miR-221-3p[§]	−3.04	**< 0.001**	**< 0.001**
miR-663b[§]	111.98	**< 0.001**	**< 0.001**	miR-222-3p[§]	−2.95	**< 0.001**	**< 0.001**
miR-21-3p[§]	4.32	**< 0.001**	**< 0.001**	miR-376c-3p[§]	−3.90	**< 0.001**	**< 0.001**
miR-615-3p[§]	26.52	**< 0.001**	**< 0.001**	miR-23b-3p	−1.75	**< 0.001**	**< 0.001**
miR-92a-3p	1.81	**< 0.001**	**< 0.001**	miR-30c-5p	−1.59	**< 0.001**	**< 0.001**
miR-425-5p[§]	2.78	**< 0.001**	**< 0.001**	miR-136-5p[§]	−6.10	**< 0.001**	**< 0.001**
miR-664a-3p	1.95	**< 0.001**	**< 0.001**	miR-27b-3p	−1.96	**< 0.001**	**< 0.001**
miR-1260a[§]	2.19	**< 0.001**	**< 0.001**	miR-451a[§]	−5.82	**< 0.001**	**< 0.001**
miR-191-5p	1.56	**< 0.001**	**< 0.001**	miR-455-3p[§]	−2.95	**< 0.001**	**< 0.001**
miR-93-5p[§]	2.05	**< 0.001**	**< 0.001**	miR-152[§]	−2.21	**< 0.001**	**< 0.001**
miR-1913[§]	2.01	**< 0.001**	**0.001**	miR-335-5p[§]	−3.24	**< 0.001**	**0.001**
miR-663a[§]	3.52	**< 0.001**	**0.001**	miR-362-3p[§]	−7.02	**< 0.001**	**0.001**
miR-484	1.66	**0.001**	**0.004**	miR-376a-3p[§]	−4.86	**< 0.001**	**0.001**
miR-7-5p[§]	4.75	**0.001**	**0.005**	miR-24-3p	−1.50	**< 0.001**	**0.001**
miR-423-5p	1.71	**0.002**	**0.010**	miR-30e-5p	−1.65	**< 0.001**	**0.001**
miR-423-3p	1.43	**0.003**	**0.012**	miR-199a-5p	−1.95	**< 0.001**	**0.001**
miR-92b-3p[§]	2.25	**0.003**	**0.015**	miR-455-5p[§]	−4.55	**< 0.001**	**0.002**
miR-182-5p[§]	2.22	**0.005**	**0.023**	miR-29a-3p	−1.51	**< 0.001**	**0.002**
miR-30d-3p[§]	2.52	**0.009**	**0.034**	miR-23a-3p	−1.38	**< 0.001**	**0.002**
miR-183-5p[§]	2.16	**0.010**	**0.038**	miR-214-5p	−4.43	**< 0.001**	**0.002**
miR-339-3p	1.63	**0.013**	**0.045**	miR-30a-3p	−2.02	**< 0.001**	**0.003**
let-7b-5p	1.44	**0.014**	**0.048**	miR-22-3p	−1.51	**< 0.001**	**0.003**

let-7d-3p	1.71	**0.014**	**0.048**	miR-130a-3p	−1.50	**0.001**	**0.004**
miR-1248	4.69	**0.015**	0.051	miR-132-3p	−3.30	**0.001**	**0.004**
miR-200b-3p	1.19	**0.015**	0.051	miR-181a-5p	−1.54	**0.001**	**0.005**
let-7a-5p	1.38	**0.016**	0.052	miR-125b-5p	−1.40	**0.001**	**0.005**
miR-130b-3p	2.18	**0.020**	0.062	miR-195-5p	−1.72	**0.001**	**0.005**
miR-103a-3p	1.26	**0.020**	0.062	miR-101-3p§	−2.07	**0.001**	**0.005**
miR-96-5p	2.66	**0.021**	0.065	miR-199a-3p	−1.76	**0.002**	**0.009**
let-7d-5p	1.27	**0.022**	0.066	miR-99a-5p	−1.41	**0.002**	**0.010**
miR-107	1.27	**0.025**	0.071	miR-181b-5p	−1.85	**0.002**	**0.012**
miR-29b-2-5p	1.58	**0.026**	0.071	miR-154-5p§	−3.28	**0.003**	**0.016**
miR-25-3p	1.34	**0.026**	0.071	miR-19b-3p	−1.51	**0.004**	**0.019**
miR-20a-5p	1.28	**0.026**	0.071	miR-30a-5p	−1.56	**0.004**	**0.019**
miR-200b-5p	1.48	**0.030**	0.079	miR-143-3p	−1.74	**0.005**	**0.020**
miR-329	3.46	**0.034**	0.084	miR-218-5p	−2.06	**0.005**	**0.020**
miR-342-5p	2.22	**0.034**	0.084	miR-16-5p	−1.37	**0.005**	**0.023**
miR-196b-5p	1.37	**0.034**	0.084	miR-338-3p	−3.10	**0.007**	**0.028**
miR-93-3p	2.24	**0.039**	0.094	miR-15a-5p	−1.47	**0.009**	**0.034**
miR-331-3p	1.50	**0.043**	0.103	miR-33a-5p	−3.21	**0.012**	**0.044**
miR-421	2.34	**0.049**	0.116	miR-502-3p	−1.89	**0.012**	**0.044**
				miR-660-5p	−2.23	**0.014**	**0.049**
				miR-149-5p	−2.01	**0.015**	**0.049**
				miR-223-3p	−1.81	**0.015**	**0.049**
				miR-199b-5p	−1.83	**0.020**	0.062
				miR-30e-3p	−1.53	**0.022**	0.065
				miR-497-5p	−1.41	**0.022**	0.065
				miR-99a-3p	−1.42	**0.023**	0.066
				miR-127-3p	−1.71	**0.025**	0.071
				miR-424-5p	−3.08	**0.025**	0.071
				miR-155-5p	−2.13	**0.027**	0.071
				miR-34a-5p	−1.39	**0.028**	0.075
				miR-125b-2-3p	−2.28	**0.033**	0.084
				miR-29b-3p	−1.29	**0.034**	0.084
				miR-26a-5p	−1.26	**0.037**	0.092
				miR-27a-3p	−1.47	**0.039**	0.095
				miR-363-3p	−1.49	**0.040**	0.095

Abbreviations: FC, fold change calculated from the mean of each group (the reciprocal number multiplied by −1 is given for downregulated miRNAs).

P values were calculated using the Wilcoxon signed-rank test and adjusted for multiple testing by the Benjamini-Hochberg (BH) method.

$P < 0.05$ was considered significant and marked in bold.

§The 29 miRNAs chosen for validation. These miRNAs were selected based on low *P*-value and high fold change in cohort 1, and were expressed in > 70% of the samples in each sample subgroup.

Supplementary Table S3: List of significantly deregulated miRNAs in primary tumor samples from patients with non-metastatic (*n* = 127) *vs.* metastatic PC (*n* = 7) in cohort 1

Upregulated in MPC	FC	*P* value	BH corrected *P* value	Downregulated in MPC	FC	*P* value	BH corrected *P* value
miR-324-5p	1.76	**0.001**	0.160	miR-133b	−2.19	**0.006**	0.160
miR-194-5p	1.99	**0.002**	0.160	miR-135a-5p	−1.88	**0.007**	0.160
miR-107	1.62	**0.002**	0.160	miR-133a	−2.11	**0.007**	0.160
miR-181b-5p	2.44	**0.003**	0.160	miR-145-3p	−1.89	**0.011**	0.204
miR-98-5p	2.02	**0.004**	0.160	miR-221-3p	−1.54	**0.027**	0.372
miR-326	2.20	**0.005**	0.160	miR-663b	−2.40	**0.030**	0.387
miR-425-5p	1.81	**0.005**	0.160	miR-155-5p	−1.08	**0.042**	0.388
miR-103a-3p	1.50	**0.006**	0.160				
miR-210	2.81	**0.011**	0.204				
miR-191-5p	1.37	**0.015**	0.258				
miR-15b-5p	2.09	**0.020**	0.308				
miR-501-5p	1.77	**0.023**	0.338				
miR-335-3p	2.11	**0.031**	0.387				
miR-532-5p	1.45	**0.033**	0.387				
miR-185-5p	2.18	**0.040**	0.388				
miR-181a-5p	1.99	**0.042**	0.388				
miR-324-3p	1.35	**0.044**	0.388				
miR-193b-3p	1.61	**0.045**	0.388				
miR-454-3p	1.83	**0.046**	0.388				
let-7i-5p	1.43	**0.047**	0.388				
miR-652-3p	1.78	**0.048**	0.388				
miR-29b-3p	1.27	**0.049**	0.388				

Abbreviations: FC, fold change calculated from the mean of each group (the reciprocal number multiplied by −1 is given for downregulated miRNAs).
P values were calculated using the Wilcoxon signed-rank test and adjusted for multiple testing by the Benjamini-Hochberg (BH) method.
$P < 0.05$ was considered significant and marked in bold.

Supplementary Table S4: List of significantly deregulated miRNAs in pT2 (*n* = 78) *vs.* pT3-4 (*n* = 49) tumors in cohort 1

Upregulated in pT3-4	FC	*P* value	BH corrected *P* value	Downregulated in pT3-4	FC	*P* value	BH corrected *P* value
miR-199b-5p	1.29	**0.012**	0.724	miR-145-5p	−1.28	**0.005**	0.724
miR-141-3p	1.29	**0.020**	0.724	miR-34a-5p	−1.19	**0.011**	0.724
miR-106b-5p	1.21	**0.022**	0.724	miR-133a	−1.40	**0.023**	0.724
miR-148a-3p	1.23	**0.024**	0.724	miR-133b	−1.27	**0.029**	0.724
miR-25-3p	1.20	**0.036**	0.724	miR-125b-2-3p	−1.24	**0.030**	0.724
miR-32-5p	1.21	**0.040**	0.724	miR-1	−1.18	**0.032**	0.724
miR-19a-3p	1.24	**0.046**	0.724	miR-326	−1.89	**0.045**	0.724

Abbreviations: FC, fold change calculated from the mean of each group (the reciprocal number multiplied by −1 is given for downregulated miRNAs); pT, pathological tumor stage.
P values were calculated using the Wilcoxon signed-rank test and adjusted for multiple testing by the Benjamini-Hochberg (BH) method.
$P < 0.05$ was considered significant and marked in bold.

Supplementary Table S5: List of significantly deregulated miRNAs in Gleason score < 7 (*n* = 60) *vs.* Gleason score ≥ 7 (*n* = 67) tumors in cohort 1

Upregulated in high Gleason score	FC	*P* value	BH corrected *P* value	Downregulated in high Gleason score	FC	*P* value	BH corrected *P* value
let-7i-5p	1.22	**0.002**	0.411	miR-664a-3p	−1.27	**0.008**	0.411
miR-185-5p	1.42	**0.016**	0.511	miR-133a	−1.48	**0.008**	0.511
miR-136-5p	2.45	**0.020**	0.511	miR-221-3p	−1.27	**0.009**	0.511
miR-106b-5p	1.36	**0.022**	0.511	miR-222-3p	−1.25	**0.010**	0.511
miR-29a-5p	1.22	**0.022**	0.511	miR-200b-5p	−1.07	**0.013**	0.588
miR-199a-5p	1.32	**0.023**	0.511	miR-1260a	−1.18	**0.016**	0.588
miR-23a-3p	1.15	**0.024**	0.511	miR-30a-3p	−1.21	**0.020**	0.588
miR-146a-5p	1.25	**0.026**	0.511	miR-21-3p	−1.36	**0.026**	0.588
let-7i-3p	1.62	**0.045**	0.588	miR-205-5p	−2.28	**0.044**	0.588
miR-26b-3p	1.16	**0.048**	0.588				

Abbreviations: FC, fold change calculated from the mean of each group (the reciprocal number multiplied by −1 is given for downregulated miRNAs).

P values were calculated using the Wilcoxon signed-rank test and adjusted for multiple testing by the Benjamini-Hochberg (BH) method.

P < 0.05 was considered significant and marked in bold.

Supplementary Table S6: List of significantly deregulated miRNAs in tumor samples from patients with (*n* = 57) *vs.* without biochemical recurrence (*n* = 70) after RP in cohort 1

Upregulated in recurrence	FC	*P* value	BH corrected *P* value	Downregulated in recurrence	FC	*P* value	BH corrected *P* value
miR-615-3p	2.86	**< 0.001**	0.060	miR-374b-5p	−1.49	**0.002**	0.132
miR-185-5p	1.54	**0.001**	0.060	miR-135a-5p	−1.37	**0.003**	0.184
miR-23a-3p	1.19	**0.007**	0.315	miR-193a-5p	−1.36	**0.008**	0.315
miR-625-3p	1.94	**0.011**	0.315	miR-222-3p	−1.18	**0.011**	0.384
let-7d-5p	1.17	**0.020**	0.481	miR-1	−1.32	**0.021**	0.481
miR-10b-5p	1.31	**0.038**	0.505	miR-133a	−1.38	**0.021**	0.481
miR-501-3p	1.56	**0.041**	0.505	miR-221-3p	−1.26	**0.022**	0.481
miR-29c-5p	1.16	**0.041**	0.505	miR-106a-5p	−1.26	**0.027**	0.500
let-7f-1-3p	1.37	**0.046**	0.538	miR-382-5p	−1.88	**0.031**	0.500
				miR-30d-3p	−1.66	**0.033**	0.500
				miR-204-5p	−1.28	**0.034**	0.500

Abbreviations: FC, fold change calculated from the mean of each group (the reciprocal number multiplied by −1 is given for downregulated miRNAs).

P values were calculated using the Wilcoxon signed-rank test and adjusted for multiple testing by the Benjamini-Hochberg (BH) method.

P < 0.05 was considered significant and marked in bold.

Supplementary Table S7: MicroRNAs selected for validation from the NM *vs.* PC comparison in cohort 1. Results from both the training and validation cohort are shown

	Cohort 1 (13 NM *vs.* 134 PC)			Cohort 2 (19 NM *vs.* 138 PC)		
Upregulated in PC	FC	*P* value	BH corrected *P* value	FC	*P* value	BH corrected *P* value
miR-375	3.31	**< 0.001**	**< 0.001**	2.46	**< 0.001**	**< 0.001**
miR-200c-3p	2.74	**< 0.001**	**< 0.001**	1.84	**< 0.001**	**< 0.001**
miR-663b	111.98	**< 0.001**	**< 0.001**	4.37	**0.022**	**0.030**
miR-21-3p	4.32	**< 0.001**	**< 0.001**	1.77	**0.004**	**0.008**

miR-615-3p	26.52	**< 0.001**	**< 0.001**	15.47	**< 0.001**	**< 0.001**
miR-425-5p	2.78	**< 0.001**	**< 0.001**	2.59	**< 0.001**	**< 0.001**
miR-1260a	2.19	**< 0.001**	**< 0.001**	1.13	0.304	0.339
miR-93-5p	2.05	**< 0.001**	**< 0.001**	1.66	**< 0.001**	**< 0.001**
miR-1913	2.01	**< 0.001**	**0.001**	1.28	**0.017**	**0.024**
miR-663a	3.52	**< 0.001**	**0.001**	3.99	**0.002**	**0.005**
miR-7-5p	4.75	**0.001**	**0.005**	1.90	0.073	0.092
miR-92b-3p	2.25	**0.003**	**0.015**	-1.56	0.212	0.256
miR-182-5p	2.22	**0.005**	**0.023**	3.43	**< 0.001**	**< 0.001**
miR-30d-3p	2.52	**0.009**	**0.034**	-1.52	0.540	0.579
miR-183-5p	2.16	**0.010**	**0.038**	2.72	**0.005**	**0.009**
**Downregulated in PC**	**FC**	***P* value**	**BH corrected *P* value**	**FC**	***P* value**	**BH corrected *P* value**
miR-205-5p	−22.39	**< 0.001**	**< 0.001**	−23.12	**< 0.001**	**< 0.001**
miR-221-3p	−3.04	**< 0.001**	**< 0.001**	−3.26	**< 0.001**	**< 0.001**
miR-222-3p	−2.95	**< 0.001**	**< 0.001**	−2.83	**< 0.001**	**< 0.001**
miR-376c-3p	−3.90	**< 0.001**	**< 0.001**	−2.62	**0.001**	**0.001**
miR-136-5p	−6.10	**< 0.001**	**< 0.001**	−2.40	**0.012**	**0.018**
miR-451a	−5.82	**< 0.001**	**< 0.001**	1.10	0.851	0.851
miR-455-3p	−2.95	**< 0.001**	**< 0.001**	−3.48	**< 0.001**	**< 0.001**
miR-152	−2.21	**< 0.001**	**< 0.001**	−1.81	**< 0.001**	**0.001**
miR-335-5p	−3.24	**< 0.001**	**0.001**	−1.96	**0.011**	**0.017**
miR-362-3p	−7.02	**< 0.001**	**0.001**	1.34	0.743	0.769
miR-376a-3p	−4.86	**< 0.001**	**0.001**	−1.83	**0.028**	**0.037**
miR-455-5p	−4.55	**< 0.001**	**0.002**	−3.33	**0.004**	**0.008**
miR-101-3p	−2.07	**0.001**	**0.005**	−1.28	0.301	0.339
miR-154-5p	−3.28	**0.003**	**0.016**	−3.33	**0.004**	**0.008**

Abbreviations: FC, fold change calculated from the mean of each group (the reciprocal number multiplied by −1 is given for downregulated miRNAs).

P values were calculated using the Wilcoxon signed-rank test and adjusted for multiple testing by the Benjamini-Hochberg (BH) method.

P < 0.05 was considered significant and marked in bold.

Supplementary Table S8: MicroRNAs selected for validation from the non-metastatic *vs.* metastatic PC comparison in cohort 1 results from both the training and validation cohort are shown

	Cohort 1 (127 PC *vs.* 7 MPC)			Cohort 2 (112 PC *vs.* 26 MPC)		
**Upregulated in MPC**	**FC**	***P* value**	**BH corrected *P* value**	**FC**	***P* value**	**BH corrected *P* value**
miR-181b-5p	2.44	**0.004**	0.160	−1.48	0.594	0.775
miR-98-5p	2.02	**0.005**	0.160	−2.47	< 0.001	0.002
miR-326	2.20	**0.005**	0.160	−1.70	0.918	0.922
miR-210	2.81	**0.011**	0.204	1.22	0.503	0.775
miR-15b-5p	2.09	**0.022**	0.308	1.38	0.094	0.187
miR-335-3p	2.11	**0.033**	0.387	1.49	0.620	0.775
miR-185-5p	2.18	**0.041**	0.388	1.49	**0.036**	0.090
**Downregulated in MPC**	**FC**	***P* value**	**BH corrected *P* value**	**FC**	***P* value**	**BH corrected *P* value**
miR-133b	−2.19	**0.006**	0.160	−3.62	**< 0.001**	**0.002**
miR-133a	−2.11	**0.007**	0.160	−2.76	**0.001**	**0.003**
miR-663b	−2.40	**0.030**	0.387	2.50	0.926	0.922

Abbreviations: FC, fold change calculated from the mean of each group (the reciprocal number multiplied by -1 is given for

downregulated miRNAs); MPC, metastatic prostate cancer; PC, non-metastatic prostate cancer.
P values were calculated using the Wilcoxon signed-rank test and adjusted for multiple testing by the Benjamini-Hochberg (BH) method.
P < 0.05 was considered significant and marked in bold.

Supplementary Table S9: MicroRNAs selected for validation from the pT2 *vs.* pT3-4 comparison in cohort 1. Results from both the training and validation cohort are shown

	Cohort 1 (78 pT2 *vs.* 49 pT3-4)			Cohort 2 (66 pT2 *vs.* 46 pT3-4)		
**Upregulated in pT3-4**	**FC**	***P* value**	**BH corrected *P* value**	**FC**	***P* value**	**BH corrected *P* value**
miR-199b-5p	1.29	**0.013**	0.724	1.05	0.261	0.392
miR-141-3p	1.29	**0.022**	0.724	1.05	0.892	0.892
**Downregulated in pT3-4**	**FC**	***P* value**	**BH corrected *P* value**	**FC**	***P* value**	**BH corrected *P* value**
miR-145-5p	−1.28	**0.005**	0.724	−1.23	0.141	0.282
miR-133a	−1.40	**0.023**	0.724	−1.29	**0.046**	0.185
miR-133b	−1.27	**0.029**	0.724	−1.26	0.062	0.185
miR-326	−1.89	**0.045**	0.724	1.61	0.369	0.442

Abbreviations: FC, fold change calculated from the mean of each group (the reciprocal number multiplied by −1 is given for downregulated miRNAs), pT, pathological tumor stage.
P values were calculated using the Wilcoxon signed-rank test and adjusted for multiple testing by the Benjamini-Hochberg (BH) method.
P < 0.05 was considered significant and marked in bold.

Supplementary Table S10: MicroRNAs selected for validation from the low (< 7) *vs.* high (≥ 7) Gleason score comparison in cohort 1. Results from both the training and validation cohort are shown

	Cohort 1 (60 low *vs.* 67 high GS)			Cohort 2 (43 low *vs.* 69 high GS)		
**Upregulated in high GS**	**FC**	***P* value**	**BH corrected *P* value**	**FC**	***P* value**	**BH corrected *P* value**
miR-185-5p	1.42	**0.016**	0.511	1.16	0.651	0.869
miR-136-5p	2.45	**0.020**	0.511	−1.51	0.969	0.983
miR-106b-5p	1.36	**0.022**	0.511	1.02	0.183	0.366
miR-199a-5p	1.32	**0.023**	0.511	−1.22	0.841	0.983
miR-146a-5p	1.25	**0.026**	0.511	−1.66	0.051	0.122
let-7i-3p	1.62	**0.045**	0.588	−1.17	0.983	0.983
**Downregulated in high GS**	**FC**	***P* value**	**BH corrected *P* value**	**FC**	***P* value**	**BH corrected *P* value**
miR-664a-3p	−1.27	**0.003**	0.411	1.02	0.490	0.734
miR-133a	−1.48	**0.008**	0.511	−1.85	**< 0.001**	**0.001**
miR-221-3p	−1.27	**0.013**	0.511	−1.77	**0.001**	**0.002**
miR-222-3p	−1.25	**0.026**	0.511	−1.66	**< 0.001**	**0.002**
miR-21-3p	−1.36	**0.047**	0.588	−1.21	0.358	0.614
miR-205-5p	−2.28	**0.047**	0.588	−3.98	**0.003**	**0.008**

Abbreviations: FC, fold change calculated from the mean of each group (the reciprocal number multiplied by −1 is given for downregulated miRNAs) GS, Gleason score.
P values were calculated using the Wilcoxon signed-rank test and adjusted for multiple testing by the Benjamini-Hochberg (BH) method.
P < 0.05 was considered significant and marked in bold.

Supplementary Table S11: List of microRNAs selected for validation from the comparison of patients with *vs.* without biochemical recurrence after RP in cohort 1. Results from both the training and validation cohort are shown

	Cohort 1 (70 wo. BCR *vs.* 57 w. BCR)			Cohort 2 (*n* = 62 wo. BCR *vs.* 50 w. BCR)		
**Upregulated in recurrence**	**FC**	***P* value**	**BH corrected *P* value**	**FC**	***P* value**	**BH corrected *P* value**
miR-615-3p	2.86	**< 0.001**	0.060	1.64	0.124	0.360
miR-185-5p	1.54	**0.001**	0.060	1.12	0.631	0.775
miR-23a-3p	1.19	**0.007**	0.315	−1.16	0.215	0.421
miR-625-3p	1.94	**0.011**	0.315	−1.79	0.050	0.360
let-7d-5p	1.17	**0.020**	0.481	−1.09	0.531	0.721
miR-10b-5p	1.31	**0.038**	0.505	1.20	0.249	0.421
miR-29c-5p	1.16	**0.041**	0.505	−1.25	0.652	0.775
miR-501-3p	1.56	**0.041**	0.505	−1.52	0.237	0.421
let-7f-1-3p	1.37	**0.046**	0.538	1.10	0.527	0.721
**Downregulated in recurrence**	**FC**	***P* value**	**BH corrected *P* value**	**FC**	***P* value**	**BH corrected *P* value**
miR-374b-5p	−1.49	**0.002**	0.132	−1.06	0.861	0.908
miR-135a-5p	−1.37	**0.003**	0.184	−1.20	0.133	0.360
miR-193a-5p	−1.36	**0.008**	0.315	1.14	**0.266**	0.421
miR-222-3p	−1.18	**0.011**	0.384	−1.40	**0.027**	0.171
miR-1	−1.32	**0.021**	0.481	−1.79	**0.021**	0.171
miR-133a	−1.38	**0.021**	0.481	−1.61	**0.007**	0.140
miR-221-3p	−1.26	**0.022**	0.481	−1.53	**0.049**	0.185
miR-106a-5p	−1.26	**0.027**	0.500	−1.05	0.704	0.786
miR-382-5p	−1.88	**0.031**	0.500	1.17	0.953	0.953
miR-30d-3p	−1.66	**0.033**	0.500	−1.46	0.160	0.380
miR-204-5p	−1.28	**0.034**	0.500	−2.33	**0.039**	0.185

Abbreviations: FC, fold change calculated from the mean of each group (the reciprocal number multiplied by −1 is given for downregulated miRNAs); BCR, biochemical recurrence after radical prostatectomy (RP).
P values were calculated using the Wilcoxon signed-rank test and adjusted for multiple testing by the Benjamini-Hochberg (BH) method.
$P < 0.05$ was considered significant and marked in bold.

Supplementary Table S12: Successfully validated miRNAs from the following comparisons: Non-metastatic versus metastatic PC, organ-confined T2 versus non organ-confined T3-4 stage, low (< 7) versus high (≥ 7) Gleason score, and no recurrence versus recurrence. Expression data from both the training and the two validation cohorts are shown

Sample groups compared	Cohort 1 (training)				Cohort 2 (validation)				Cohort 3 (external validation)			
	FC	*P* value	BH corrected *P* value	AUC (95% CI)	FC	*P* value	BH corrected *P* value	AUC (95% CI)	FC	*P* value	BH corrected *P* value	AUC (95% CI)
Upregulated in MPC	***127 non-metastatic PC vs. 7 MPC***				***112 non-metastatic PC vs. 26 MPC***				***99 non-metastatic PC vs. 14 MPC***			
miR-185-5p	2.18	**0.041**	0.388	0.73 (0.51–0.95)	1.49	**0.036**	0.068	0.63 (0.51–0.76)	1.49	**0.004**	**0.004**	0.74 (0.55–0.93)
Downregulated in MPC												
miR-133b	−3.99	**0.006**	0.160	0.81 (0.59–1.00)	−3.62	**< 0.001**	**0.002**	0.72 (0.60–0.85)	−20.8	**< 0.001**	**< 0.001**	0.97 (0.92–1.00)
miR-133a	−3.95	**0.007**	0.160	0.80 (0.56–1.00)	−2.76	**0.001**	**0.003**	0.71 (0.60–0.82)	−8.07	**< 0.001**	**< 0.001**	0.98 (0.93–1.00)
Downregulated in pT3-4 stages	***78 pT2 vs. 49 pT3-4***				66 pT2 vs. 46 pT3-4				***69 pT2 vs. 30 pT3-4***			
miR-133a	−1.40	**0.023**	0.724	0.62 (0.51–0.73)	−1.29	**0.046**	0.185	0.61 (0.51–0.72)	−1.35	**0.013**	**0.0128**	0.66 (0.54–0.77)
Downregulated in high Gleason score	***Gleason score: 60 low vs. 67 high***				***Gleason score: 43 low vs. 69 high***				***Gleason score: 32 low vs. 67 high***			
miR-133a	−1.48	**0.008**	0.511	0.64 (0.54–0.73)	−1.85	**< 0.001**	**0.001**	0.73 (0.63–0.82)	−1.27	**0.025**	0.100	0.64 (0.52–0.76)
miR-221-3p	−1.27	**0.013**	0.511	0.63 (0.53–0.73)	−1.77	**0.001**	**0.002**	0.70 (0.60–0.80)	−1.23	0.220	0.220	0.52 (0.40–0.64)
miR-222-3p	−1.25	**0.026**	0.511	0.62 (0.52–0.71)	−1.66	**< 0.001**	**0.002**	0.70 (0.61–0.80)	−1.24	0.138	0.183	0.56 (0.44–0.68)
miR-205-5p	−2.28	**0.047**	0.588	0.60 (0.50–0.70)	−3.98	**0.003**	**0.008**	0.67 (0.56–0.78)	−1.67	0.093	0.183	0.61 (0.48–0.73)
Downregulated in recurrence	***70 no recurrence vs. 57 recurrence***				***62 no recurrence vs. 50 recurrence***				***74 no recurrence vs. 25 recurrence***			
miR-222-3p	−1.18	**0.011**	0.384	0.63 (0.53–0.73)	−1.40	**0.027**	0.171	0.62 (0.52–0.73)	−1.05	0.690	0.690	0.53 (0.39–0.67)
miR-1	−1.32	**0.021**	0.481	0.62 (0.52–0.72)	−1.79	**0.021**	0.171	0.63 (0.52–0.73)	−1.41	**0.045**	0.112	0.63 (0.51–0.76)
miR-133a	−1.38	**0.021**	0.481	0.62 (0.52–0.72)	−1.61	**0.007**	0.140	0.65 (0.55–0.75)	−1.14	0.618	0.690	0.53 (0.39–0.67)
miR-221-3p	−1.26	**0.022**	0.481	0.62 (0.52–0.72)	−1.53	**0.049**	0.185	0.61 (0.50–0.71)	−1.42	**0.029**	0.112	0.65 (0.52–0.77)
miR-204-5p	−1.28	**0.034**	0.500	0.61 (0.51–0.71)	−2.33	**0.039**	0.185	0.61 (0.51–0.72)	−1.16	0.387	0.644	0.56 (0.42–0.70)

Abbreviations: AUC, area under the curve; CI, confidence interval; FC, fold change calculated from the mean of each group (the reciprocal number multiplied by −1 is given for downregulated miRNAs); MPC, metastatic prostate cancer (primary tumor tissue from patients with metastatic or/and castration refractory PC); PC, prostate cancer; pT, pathological T-stage.

P values were calculated using Wilcoxon signed-rank test, and adjusted for multiple testing by the Benjamini-Hochberg (BH) method; $P < 0.05$ was considered significant and marked in bold

Supplementary Table S13: The 13 miRNAs used in at least 70% of the cross validation loops when building the diagnostic miRNA classifier. Results from pairwise comparisons of NM *vs.* PC samples in cohorts 1 and 2

	Cohort 1 (13 NM *vs.* 134 PC)				Cohort 2 (19 NM *vs.* 138 PC)			
**Upregulated in PC**	**FC**	***P* value**	**BH corrected *P* value**	**AUC (95% CI)**	**FC**	***P* value**	**BH corrected *P* value**	**AUC (95% CI)**
miR-663b	111.98	**< 0.001**	**< 0.001**	0.94 (0.90–0.99)	4.37	**0.022**	**0.028**	0.66 (0.53–0.79)
miR-615-3p	26.52	**< 0.001**	**< 0.001**	0.90 (0.85–0.96)	15.47	**< 0.001**	**< 0.001**	0.83 (0.74–0.93)
miR-21-3p	4.32	**< 0.001**	**< 0.001**	0.91 (0.84–0.98)	1.77	**0.004**	**0.008**	0.70 (0.60–0.81)
miR-1260a	2.19	**< 0.001**	**< 0.001**	0.87 (0.75–0.99)	1.13	0.304	0.304	0.57 (0.45–0.69)
miR-93-5p	2.05	**< 0.001**	**< 0.001**	0.84 (0.77–0.92)	1.66	**< 0.001**	**< 0.001**	0.84 (0.77–0.90)
miR-664a-3p	1.95	**< 0.001**	**< 0.001**	0.89 (0.82–0.96)	2.05	**0.008**	**0.014**	0.69 (0.56–0.81)
miR-92a-3p	1.81	**< 0.001**	**< 0.001**	0.90 (0.85–0.95)	1.32	**0.011**	**0.015**	0.68 (0.57–0.79)
**Downregulated in PC**	**FC**	***P* value**	**BH corrected *P* value**	**AUC (95% CI)**	**FC**	***P* value**	**BH corrected *P* value**	**AUC (95% CI)**
miR-221-3p	–3.04	**< 0.001**	**< 0.001**	0.95 (0.90–1.00)	−3.26	**< 0.001**	**< 0.001**	0.88 (0.80–0.96)
miR-222-3p	–2.95	**< 0.001**	**< 0.001**	0.92 (0.83–1.00)	–2.83	**< 0.001**	**< 0.001**	0.88 (0.81–0.95)
miR-27b-3p	–1.96	**< 0.001**	**< 0.001**	0.86 (0.77–0.96)	–1.51	**0.001**	**0.003**	0.73 (0.63–0.83)
miR-23b-3p	–1.75	**< 0.001**	**< 0.001**	0.92 (0.86–0.97)	–1.56	**< 0.001**	**< 0.001**	0.77 (0.67–0.86)
miR-30c-5p	–1.59	**< 0.001**	**< 0.001**	0.87 (0.74–0.99)	–1.23	0.119	0.128	0.61 (0.50–0.73)
miR-29a-3p	–1.51	**< 0.001**	**< 0.001**	0.81 (0.65–0.97)	–1.26	**0.024**	**0.028**	0.66 (0.54–0.78)

Abbreviations: AUC, area under the curve from ROC curve analysis; CI, confidence interval; FC: fold change calculated from the mean of each group (the reciprocal number multiplied by –1 is given for downregulated miRNAs).
P values were calculated using the Wilcoxon signed-rank test and adjusted for multiple testing by the Benjamini-Hochberg (BH) method.
$P < 0.05$ was considered significant and marked in bold.

Supplementary Table S14: Diagnostic performance of the 13-miRNA diagnostic classifier as compared to top candidate single diagnostic miRNAs (from Table 2) in the discovery and validation cohort

	Cohort 1 (13 NM *vs.* 134 PC)				Cohort 2 (19 NM *vs.* 138 PC)			
	***P* value**[#]	**Correctly classified (%)***	**Sens (%)***	**Spec (%)***	***P* value**[#]	**Correctly classified (%)***	**Sens (%)***	**Spec (%)***
**13-miRNA diagnostic classifier**	< 0.001	95.9	96.3	92.3	< 0.001	86.0	87.7	73.7
**Upregulated in PC**								
miR-375	< 0.001	88.4	88.1	92.3	< 0.001	72.6	72.5	73.7
miR-663b	< 0.001	85.7	85.1	92.3	0.036	58.6	56.5	73.7
miR-615-3p	< 0.001	83.0	82.1	92.3	< 0.001	81.5	82.6	73.7
miR-425-5p	< 0.001	72.8	70.9	92.3	< 0.001	82.8	84.1	73.7
miR-663a	0.001	42.2	37.3	92.3	0.006	62.4	60.9	73.7
miR-182-5p	0.023	42.2	37.3	92.3	< 0.001	75.2	75.4	73.7
miR-183-5p	0.038	52.4	48.5	92.3	0.011	59.2	57.3	73.7
**Downregulated in PC**								
miR-205-5p	< 0.001	89.1	88.8	92.3	< 0.001	94.3	97.1	73.7
miR-221-3p	< 0.001	88.4	88.1	92.3	< 0.001	82.2	83.3	73.7
miR-222-3p	< 0.001	81.6	80.6	92.3	< 0.001	77.7	78.3	73.7
miR-376c-3p	< 0.001	78.2	76.9	92.3	0.002	66.2	65.2	73.7
miR-136-5p	< 0.001	76.2	74.6	92.3	0.021	61.8	60.1	73.7

miR-455-3p	< 0.001	59.2	56.0	92.3	< 0.001	72.0	71.7	73.7
miR-455-5p	0.002	46.3	41.8	92.3	0.010	58.0	55.8	73.7
miR-154-5p	0.016	34.0	28.4	92.3	0.010	62.4	60.9	73.7

Abbreviations: PC, prostate cancer; Sens, sensitivity of miRNA for detecting PC; Spec, specificity of miRNA for detecting PC. #*P* values for single miRNAs were calculated using the Wilcoxon signed-rank test and adjusted for multiple testing by the Benjamini-Hochberg (BH) method; The diagnostic classifier was tested using a χ^2 test. $P < 0.05$ was considered significant. *To directly compare the diagnostic performance of single candidate miRNAs with the 13-miRNA diagnostic classifier, their specificity was fixed by ROC curve analysis to match the specificity of the 13-miRNA diagnostic classifier in each cohort.

Supplementary Table S15: Univariate Cox regression analyses of biochemical recurrence-free survival time

Variable	Characteristics	RP cohort 1, *n* = 126, 56 with recurrence				RP cohort 2, *n* = 110, 49 with recurrence				RP cohort 3, *n* = 99, 25 with recurrence			
		HR (95% CI)	*P* value	BH corrected *P* value	C–index[a]	HR (95% CI)	*P* value	BH corrected *P* value	C–index[a]	HR (95% CI)	*P* value	BH corrected *P* value	C–index[a]
Age at diagnosis	Continuous	1.00 (0.94–1.05)	0.858	0.815	0.53	0.97 (0.93–1.03)	0.319	0.425	0.53	1.03 (0.98–1.10)	0.278	0.379	0.56
Tumor stage	pT2a-c *vs.* pT3a-c	3.12 (1.81–5.36)	**< 0.001**	**0.001**	0.64	3.00 (1.69–5.30)	**< 0.001**	**0.001**	0.64	4.05 (1.80–9.12)	**0.001**	**0.005**	0.68
Gleason score *	< 7 *vs.* ≥ 7	2.72 (1.51–4.93)	**0.001**	**0.010**	0.61	2.42 (1.23–4.73)	**0.010**	**0.032**	0.59	–	–	–	–
Surgical margin status	Negative *vs.* positive	2.73 (1.59–4.70)	**< 0.001**	**0.001**	0.63	3.37 (1.89–6.00)	**< 0.001**	**0.001**	0.64	3.81 (1.70–8.54)	**0.001**	**0.005**	0.63
Preoperative PSA	Continuous	1.05 (1.02–1.08)	**< 0.001**	**0.001**	0.62	1.05 (1.03–1.07)	**< 0.001**	**0.001**	0.72	1.09 (1.06–1.13)	**< 0.001**	**0.002**	0.66
miR-374b-5p	Continuous	0.72 (0.60–0.86)	**< 0.001**	**0.005**	0.63	1.02 (0.83–1.25)	0.831	0.878	0.55	0.57 (0.31–1.03)	0.062	**0.133**	0.62
miR-23a-3p	Continuous	2.57 (1.43–4.63)	**0.002**	**0.017**	0.63	0.80 (0.56–1.13)	0.205	0.298	0.54	1.33 (0.60–2.94)	0.476	0.549	0.50
miR-625-3p	Continuous	1.22 (1.06–1.40)	**0.006**	**0.043**	0.57	0.92 (0.82–1.04)	0.172	0.278	0.56	0.71 (0.41–1.24)	0.227	0.378	0.53
miR-615-3p	Continuous	1.16 (1.04–1.28)	**0.007**	**0.044**	0.63	1.04 (0.94–1.16)	0.439	0.540	0.53	1.00 (0.78–1.27)	0.973	0.973	0.50
miR-185-5p	Continuous	1.47 (1.10–1.97)	**0.009**	**0.050**	0.64	1.06 (0.85–1.33)	0.610	0.697	0.52	4.59 (1.52–13.90)	**0.007**	**0.021**	0.65
miR-133a	Continuous	0.80 (0.67–0.96)	**0.017**	**0.085**	0.57	0.78 (0.66–0.92)	**0.003**	**0.012**	0.62	0.74 (0.48–1.14)	0.174	0.326	0.57
miR-193a-5p	Continuous	0.78 (0.62–0.97)	**0.025**	**0.114**	0.60	0.98 (0.78–1.24)	0.878	0.878	0.52	1.43 (0.65–3.16)	0.374	0.468	0.57
miR-221-3p	Continuous	0.68 (0.49–0.96)	**0.029**	**0.121**	0.55	0.83 (0.69–0.98)	**0.033**	**0.075**	0.56	0.59 (0.41–0.86)	**0.005**	**0.019**	0.67
miR-326	Continuous	0.90 (0.82–0.99)	**0.032**	**0.123**	0.57	0.91 (0.84–0.99)	**0.023**	**0.061**	0.61	1.46 (0.76–2.83)	0.256	0.379	0.54
miR-10b-5p	Continuous	1.28 (1.00–1.63)	**0.046**	**0.163**	0.57	1.26 (0.93–1.71)	0.135	0.270	0.53	1.12 (0.65–1.90)	0.690	0.739	0.50
miR-30d-3p	Continuous	0.89 (0.79–1.00)	**0.049**	**0.163**	0.57	0.91 (0.81–1.03)	0.174	0.278	0.55	1.79 (1.09–2.92)	**0.020**	**0.050**	0.68

Significant miRNAs from RP cohort 1 were tested in RP cohorts 2 and 3

This Table lists 11 miRNA that were significantly associated with biochemical recurrence-free survival time in RP cohort 1. These miRNAs were identified from a list of 45 prognostic candidate miRNAs identified as deregulated in pT2 vs. pT3-4, low *vs.* high Gleason score, and/or recurrent *vs.* non-recurrent tumors in cohort 1 (Supplementary Table S4–S6).

Abbreviations: CI, confidence Interval; HR, hazard ratio, PSA, prostate specific antigen; RP, radical prostatectomy.

[a]Predictive accuracy, estimated by Harrell's concordance index (C-index).

[*]Gleason score was excluded from analysis in RP cohort 3, because the low Gleason score group (< 7) had no events.

Significant *P* values ($P < 0.05$) are marked in bold. *P* values of individual miRNAs were adjusted for multiple testing by the Benjamini-Hochberg (BH) method, FDR < 0.2 was considered significant (marked in bold).

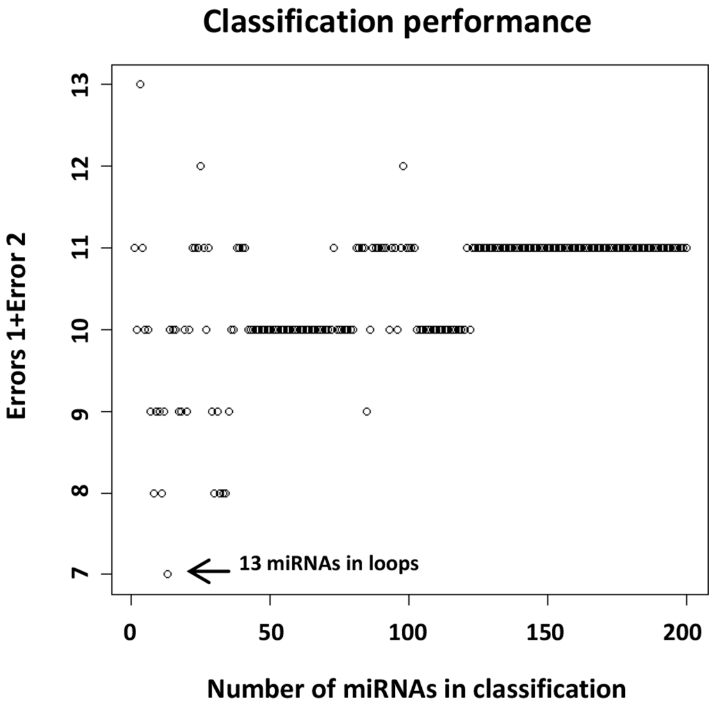

Supplementary Figure S1: Number of classification errors (misclassified in group 1 + misclassified in group 2) *vs.* number of genes used in cross validation loops. We used a LOOCV maximum likelihood classifier approach [1] to build a diagnostic classifier, which was trained on 13 NM and 134 PC samples in cohort 1. Classifier performance was tested using 1–200 genes in cross-validation loops. The lowest number of misclassifications of nonmalignant (group 1) and PC (group 2) samples was obtained by using 13 miRNAs in the loops (marked by arrow). The final 13-miRNA diagnostic classifier included miR-663b, miR-615-3p, miR-21-3p, miR-1260a, miR-93-5p, miR-664a-3p, miR-92a-3p, miR-221-3p, miR-222-3p, miR-27b-3p, miR-23b-3p, miR-30c-5p and miR-29a-3p, all of which were used in at least 70% of the cross-validations loops.

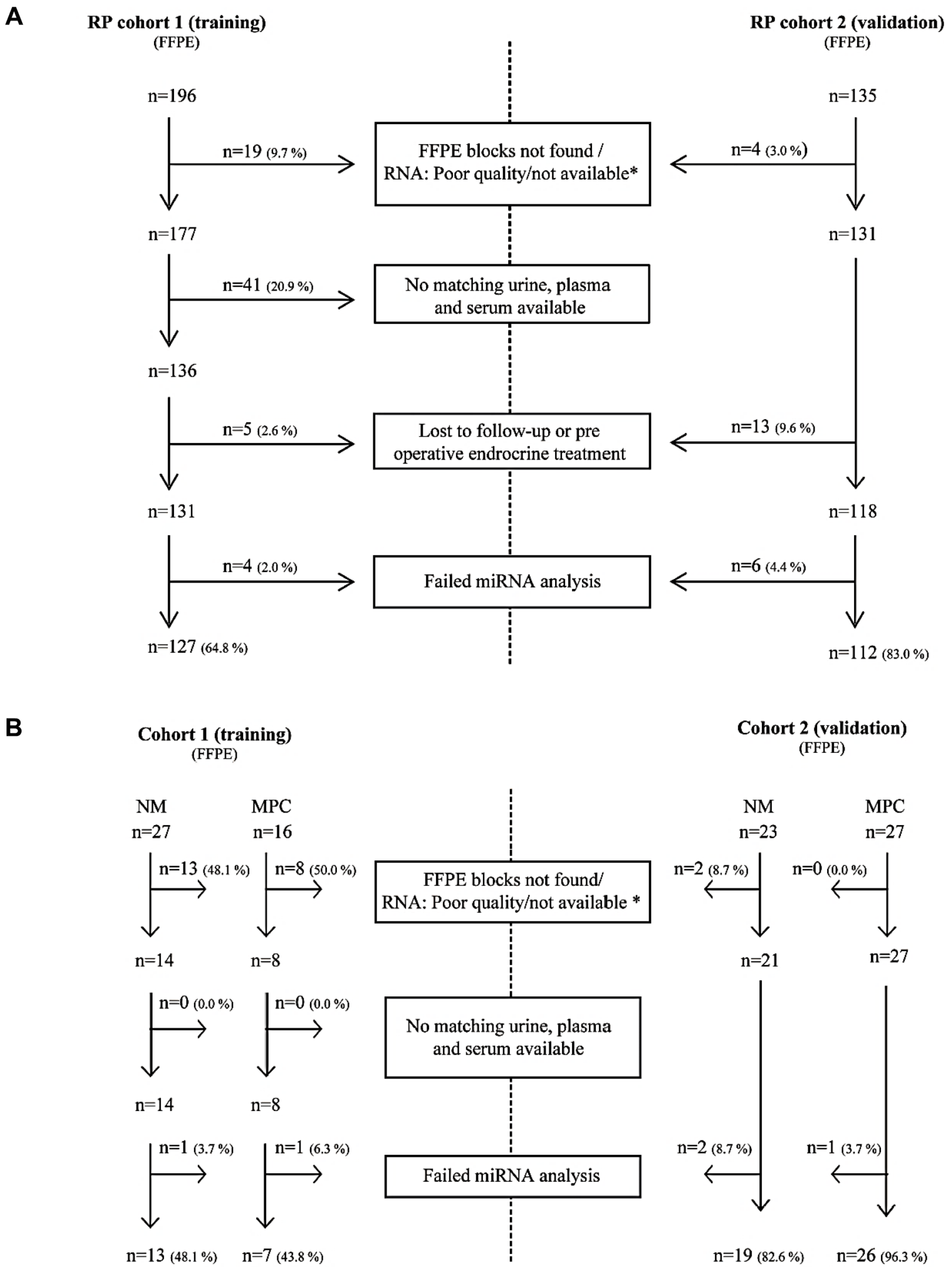

Supplementary Figure S2: Flow charts of inclusion/exclusion criteria. (**A**) Radical prostatectomy (RP) cohorts 1 and 2. (**B**) Non-malignant (NM) and metastatic PC samples used in cohorts 1 and 2. * RNA was used up in previous studies. AN, adjacent non-malignant prostate; MPC, metastatic prostate cancer (primary tumor tissue from patients with metastatic PC); FFPE, formalin-fixed and paraffin-embedded.

SUPPLEMENTARY METHODS

MiRNA profiling

MicroRNA expression profiling (all reagents from Exiqon) was performed at Exiqon A/S, Vedbaek, Denmark, using the miRCURY LNA™ Universal RT microRNA PCR platform. In brief, 40 ng total RNA was reverse transcribed in 40 µl reactions using the miRCURY LNA™ microRNA PCR, Polyadenylation and cDNA synthesis kit II. cDNA was diluted 100× and analyzed in 10 µl PCR reactions. For cohort 1 (training), relative expression levels of 752 miRNAs were analyzed using microRNA Ready-to-Use PCR, Human panel I + II, V3.R, in 384-well PCR plates. For cohort 2 (validation), 94 selected miRNAs (including normalization gene miR-151a-5p) were analyzed using a miRCURY LNA™ Universal RT Pick-&-Mix microRNA PCR panel (4 × 96 in 384-well, Ready-to-Use). Negative controls (no template in reverse transcription reaction) were run in parallel. Amplification was performed using the LightCycler® 480 Real-Time PCR System (Roche) and ExiLENT SYBR® Green master mix. Amplification curves were analyzed using the Roche LC software for determination of quantification cycle (Cq) values by the 2nd derivative method and for melting curve analyses. MicroRNAs detected at < 3 Cq below the negative control or for which Cq values exceeded 37 in all samples were excluded from further analyses (cohort 1: 97 miRNAs, cohort 2: no miRNAs).

For cohort 1 (training), data was normalized to the global mean (i.e. mean for all miRNAs detected in all samples; here $n = 61$), previously reported as the best normalization method for qRT-PCR data involving numerous assays [4]. In cohort 2 (validation), miRNA levels were normalized to miR-151a-5p, identified by the NormFinder algorithm [5] as an optimally stable single normalization gene in both cohorts 1 and 2. The two normalization strategies gave highly similar results in terms of top differentially expressed miRNAs as well as in the overall ranking of miRNAs in cohort 1 (data not shown), supporting the validity of miR-151a-5p as normalization gene in the validation study (cohort 2) with fewer miRNAs tested. Normalization was done according to the formula $\Delta Cq = Cq_{\text{Normalisation factor}} - Cq_{\text{miRNA}}$. Differences in expression levels were calculated as $\Delta\Delta Cq = \Delta Cq_{\text{group 1}} - \Delta Cq_{\text{group 2}}$. To convert this to fold change, the formula $2^{\Delta\Delta Cq}$ was used. The reciprocal number, multiplied by a factor of −1, was used for downregulated miRNAs.

REFERENCES

1. Dyrskjot L, Thykjaer T, Kruhoffer M, Jensen JL, Marcussen N, Hamilton-Dutoit S, Wolf H, Orntoft TF. Identifying distinct classes of bladder carcinoma using microarrays. Nat Genet. 2003; 33:90–96.
2. Taylor BS, Schultz N, Hieronymus H, Gopalan A, Xiao Y, Carver BS, Arora VK, Kaushik P, Cerami E, Reva B, Antipin Y, Mitsiades N, Landers T, et al. Integrative genomic profiling of human prostate cancer. Cancer Cell. 2010; 18:11–22.
3. Hieronymus H, Schultz N, Gopalan A, Carver BS, Chang MT, Xiao Y, Heguy A, Huberman K, Bernstein M, Assel M, Murali R, Vickers A, Scardino PT, et al. Copy number alteration burden predicts prostate cancer relapse. Proc Natl Acad Sci U S A. 2014; 111:11139–11144.
4. Mestdagh P, Van Vlierberghe P, De Weer A, Muth D, Westermann F, Speleman F, Vandesompele J. A novel and universal method for microRNA RT-qPCR data normalization. Genome Biol. 2009; 10:R64.
5. Andersen CL, Jensen JL, Orntoft TF. Normalization of real-time quantitative reverse transcription-PCR data: a model-based variance estimation approach to identify genes suited for normalization, applied to bladder and colon cancer data sets. Cancer research. 2004; 64:5245–5250.
